# Fully Distributed Monitoring Architecture Supporting Multiple Trackees and Trackers in Indoor Mobile Asset Management Application

**DOI:** 10.3390/s140305702

**Published:** 2014-03-21

**Authors:** Seol Young Jeong, Hyeong Gon Jo, Soon Ju Kang

**Affiliations:** School of Electronics Engineering, College of IT Engineering, Kyungpook National University, 80 Daehakro, Bukgu, Daegu, Korea; E-Mails: snowflower@ee.knu.ac.kr (S.Y.J.); tsana@ee.knu.ac.kr (H.G.J.)

**Keywords:** indoor mobile asset management, real-time location service, multiple-target tracking, self-organizing overlay network

## Abstract

A tracking service like asset management is essential in a dynamic hospital environment consisting of numerous mobile assets (e.g., wheelchairs or infusion pumps) that are continuously relocated throughout a hospital. The tracking service is accomplished based on the key technologies of an indoor location-based service (LBS), such as locating and monitoring multiple mobile targets inside a building in real time. An indoor LBS such as a tracking service entails numerous resource lookups being requested concurrently and frequently from several locations, as well as a network infrastructure requiring support for high scalability in indoor environments. A traditional centralized architecture needs to maintain a geographic map of the entire building or complex in its central server, which can cause low scalability and traffic congestion. This paper presents a self-organizing and fully distributed indoor mobile asset management (MAM) platform, and proposes an architecture for multiple trackees (such as mobile assets) and trackers based on the proposed distributed platform in real time. In order to verify the suggested platform, scalability performance according to increases in the number of concurrent lookups was evaluated in a real test bed. Tracking latency and traffic load ratio in the proposed tracking architecture was also evaluated.

## 1. Introduction

Research into efficient and reliable real-time tracking services in indoor location-based service (LBS) systems have continued since it was first necessary to consider current locations of users or mobile objects [1]. A tracking service can be accomplished based on key technologies of the indoor LBS, such as locating and monitoring multiple mobile targets inside a building in real time. Capabilities for monitoring or locating people and objects in real time are particularly appropriate for applications in a healthcare environment. In the dynamic hospital environment, numerous mobile assets, such as wheelchairs and infusion pumps, are continuously relocated throughout the hospital. In addition, patients and staff also frequently move throughout the hospital. Therefore, various real-time location systems (RTLSs) for healthcare are being provided by many manufactures like AeroScout [2] or Ekahau [3]. An RTLS offers mobile asset management (MAM), including patient monitoring, equipment management, emergency management, and so on.

In these RTLS systems, such as a complex and dynamic hospital environment, tracking service requests from multiple trackees (mobile targets) and from multiple trackers (monitoring devices) can frequently occur. Therefore, an efficient distributed architecture for a dynamic environment is needed. In addition, an architecture for an indoor MAM platform should support a heterogeneous environment regardless of communication method for localization. Hence, this paper suggests a tracking service architecture based on a self-organizing and fully distributed platform. However, for a centralized platform as described above, much of the research has many problems, such as a concentration of unnecessary traffic, local traffic overflow, or system faults because an indoor environment like a hospital has many assets, patients, and staff with dynamic and frequent mobility inside a complex building.

In order to provide RTLS services simultaneously and constantly available in various parts of the hospital, it is necessary to construct an efficient indoor MAM system [4,5]. This paper proposes an overlay network infrastructure called the self-organizing service platform (SoSp), for an indoor MAM system. The self-organizing service platform router (SoSp-Router), which works as one unit of the overlay network [6] based on physical distance on top of the legacy physical network, is installed in a unit space, such as a room or corridor in a hospital building. The SoSp-Router is aware of the location of all devices and people in its unit space. The SoSp-Router also possesses information about neighboring SoSp-Routers that are connected through a physical path (*i.e.*, a door or a corridor) or in a logical neighbor grouping according to user (e.g., both house and office). Each SoSp-Router can discover the needed mobile devices using only the information about its neighbors through cooperation amongst neighboring SoSp-Routers.

The proposed architecture provides a fully distributed LBS, in that it does not have a concentration of unnecessary traffic and is less sensitive to service area expansion and an increased number of devices (both mobile devices and resource devices). Furthermore, this architecture improves the scalability of the infrastructure, because there is no need to maintain a map of the entire building or complex in the central server. Most importantly, there is no single point of failure due to local traffic overflow or system faults. This paper constitutes the SoSp overlay network infrastructure, and discusses a method of multiple mobile devices tracking based on the proposed SoSp.

The remainder of the paper is structured as follows: the basic concepts and the approach towards an indoor MAM platform are discussed in Section 2, while Section 3 presents related works. Section 4 describes the proposed indoor MAM system for a tracking service and the operation principles of the lookup engine. Section 5 presents detailed operation principles of the tracking algorithm in the proposed self-organizing distributed indoor MAM system. Section 6 introduces implementation of mobile devices and the SoSp-Router in the proposed platform and demonstrates performance evaluation through simulation and a real test bed. Finally, conclusions are drawn in Section 7.

## 2. Basic Concepts and Approach

### 2.1. Dynamic Service Scenarios for Multiple Asset Management

**Figure 1.**
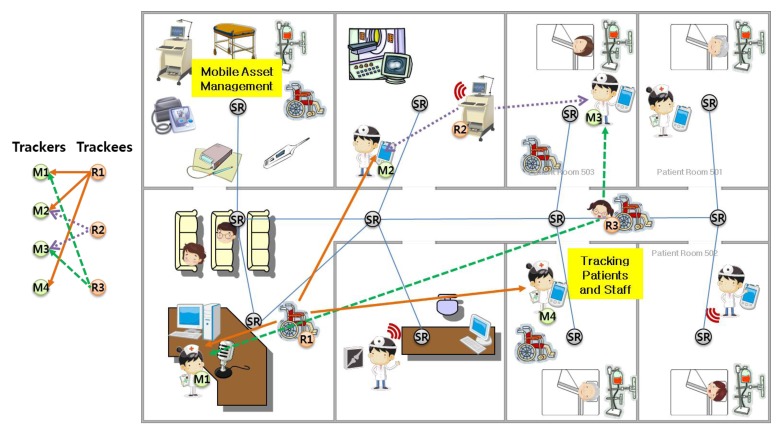
An example of an RTLS system for a hospital environment and indoor MAM services.

Figure 1 introduces an example of an indoor environment and dynamic service scenario including a tracking service. As shown in Figure 1, there are numerous pieces of hospital equipment that are either mobile or stationary, plus patients and staff with mobile devices in a hospital building.

In addition, indoor MAM for the following service examples can be requested via mobile devices like smartphones in this RTLS system:

(1) Zero-configuration based service: A mobile device user can search for the nearest wheelchair (or other hospital equipment) from his or her current position without presetting.
(2) Delay-tolerance based service: After using a local sphygmomanometer, patients or hospital staff can look up and check the medical data such as blood pressure stored at a local of equipment, anywhere and anytime regardless of current location without needing to configure any settings.
(3) Multiple mobile devices tracking service: Multiple monitoring devices can track multiple mobile devices moving in a hospital environment in real time.

In order to provide these dynamic services, the SoSp-Router works as a stationary node and represents the unit space location. Therefore, the SoSp-Router consolidates the unit's resources, as shown in Figure 1. Furthermore, the SoSp-Router manages the connection between a resource device (e.g., mobile or stationary equipment such as a wheel chair or infusion pump) and a monitoring device, such as a smartphone. The SoSp-Router can also transfer a user's private data, such as personal medical data, to particular SoSp-Routers or devices that have been appointed as a home repository by the user. One or more home repositories help establish a fully distributed environment. The primary purpose of this research is to provide indoor MAM between trackees and trackers in this overlay network.

In particular, numerous monitoring devices can request indoor MAM, like a tracking service, from several locations. Numerous dynamic targets, such as patients with frequent mobility, need real-time management inside a hospital building, and because the hospital environment is complex and dynamic configuration due to frequent movement of mobile equipment and people.

In the proposed platform, each SoSp-Router represents the position from which users request the services located within its range. In this manner, the overall network architecture is fully decentralized and localized. In addition, expansion of the service area or an increase in the number of mobile or resource devices does not increase the complexity, unlike a traditional centralized server-based architecture.

### 2.2. Platform Architecture for Indoor MAM

Indoor location-based services are becoming more prevalent owing to increases in the number of smartphone users. Furthermore, indoor LBSs have become important services that use several ubiquitous devices based on opportunistic computing. Opportunistic computing provides a great opportunity to match services to resources, exchange information, engage in cyber foraging, execute tasks remotely, and forward messages when two devices come in contact [7].

However, a system for providing an indoor LBS using a smartphone in a traditional infrastructure has many limitations. For instance, a smartphone user who wants to print from the nearest printer must search and choose the printer from the usable printer list in a network infrastructure. Furthermore, the smartphone needs to directly connect with the particular printer using a specific function such as WiFi direct or Bluetooth. Some smartphone applications must also provide a printing function in the cloud environment based on a centralized server, which leads to unnecessary overflow in the cloud server and the danger of personal information disclosure. Moreover, indoor LBS discovery differs significantly from Web service discovery that has no physical location and user context [8]. Therefore, it is necessary to develop a people-centered (or mobile device-centered) lookup mechanism and a platform architecture for the indoor LBS.

**Figure 2.**
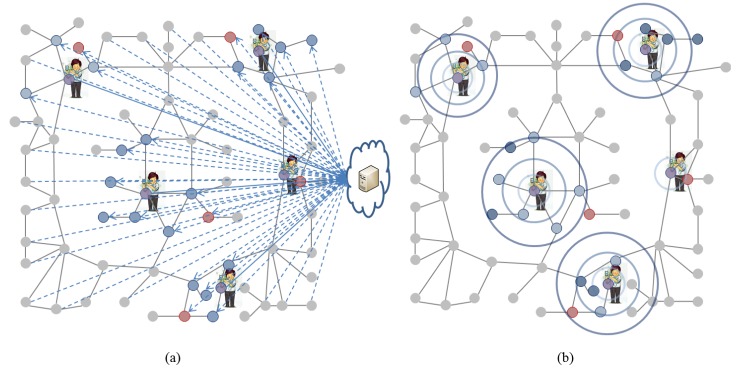
Traffic flow for a lookup service in a centralized platform and the proposed self-organizing distributed platform. (**a**) Centralized Platform; (**b**) Self-Organizing Distributed Platform.

In addition, a traditional centralized LBS system needs to maintain a geographic map of the entire building or complex in its central server, which can cause low scalability and traffic congestion. In order to solve these inherent challenges to the centralized server architecture, a location-based self-organizing fully distributed network infrastructure similar to a natural spontaneous environment with a lot of movement, like an ant or bee colony, needs to be designed. Self-organizing is a spontaneous process in which some form of global order or coordination arises out of the local interactions between the components of an initially disordered system. The self-organizing and fully distributed indoor LBS system would not need to maintain or employ a centralized map and would be dynamically adaptive to changes in the indoor infrastructure. As shown in Figure 2, since flows of traffic are mainly within the range of lookup in the indoor LBS system, the proposed distributed platform is more efficient than the traditional centralized platform. Furthermore, personal privacy would be protected without any need to forward the user's data to the central server; the requested data would only be transferred within the local range surrounding the point of the lookup activity. For example, the location-aware Cisco Unified Wireless Network (UWN) [9] has a centralized architecture.

An indoor MAM platform needs to support the various communication methods for localization of the mobile or resource devices with inherent communication protocols applicable to each one's specialized functions. Many indoor MAM systems are built in a heterogeneous environment with various communications systems, such as WiFi, Bluetooth, ZigBee, and Ethernet. In particular, these mobile devices for service in an indoor MAM should be energy-efficient with real-time localization based on continuous wireless communication.

In the proposed self-organizing distributed platform, the localization algorithm is based on two methods of Location-ID exchange & Asynchronous Message Delivery (LIDx & AMD) [10,11] wireless communication protocol that developed with our previous research: a mobile device based approach and a SoSp-Router based approach. The LIDx protocol can provide real-time localization for numerous mobile and resource devices in a complex and dynamic indoor environment, such as a hospital, warehouse, or museum.

**Figure 3.**
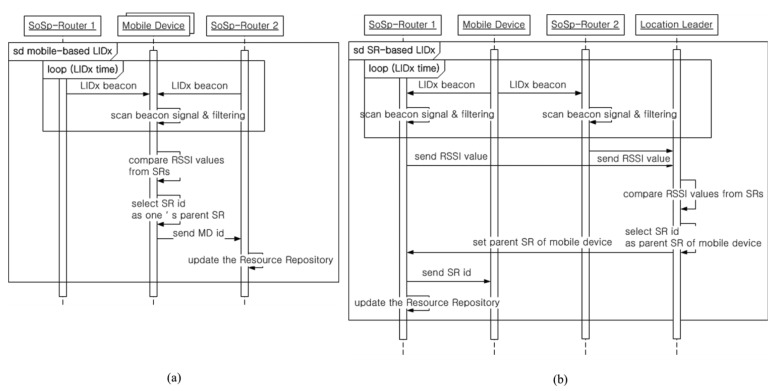
The LIDx process between the mobile devices and SoSp-Routers. (**a**) Mobile device based LIDx protocol process; (**b**) SoSp-Router based LIDx protocol process.

First, the mobile device based localization method is appropriate for a mobile device with a wireless communication protocol like ZigBee featuring low power consumption. Figure 3 describes the localization process using the LIDx protocol. This localization protocol uses a simple bidirectional communication channel between the SoSp-Router and mobile devices, so it guarantees efficient movement of mobile devices despite having to support real-time tracking of numerous mobile devices. Secondly, the SoSp-Router based localization method is used for a mobile device with a wireless communication protocol that has relatively high power consumption, such as Bluetooth or WiFi. A longer time is required to scan received packets during the LIDx period, in contrast with the time to periodically send beacon messages. Figure 3b shows the localization process via the SoSp-Router with main power.

The mobile devices periodically send a beacon message including mobile ID information to the SoSp-Router within its range. The SoSp-Routers receive the LIDx beacon message, and filter the values of the received the signal strength indication (RSSI) from the mobile devices. Each SoSp-Router sends the RSSI value to a Location Leader (one of the SoSp-Routers, selected when they are initially installed or changed). The Location Leader receiving the RSSI value between each mobile device and the SoSp-Routers compares the values and selects one of SoSp-Routers as the parent of the mobile device. The selected SoSp-Router sends the its ID to the mobile device, and updates the its Resource Repository.

In the LIDx protocol [11], a mobile device requests beaconing to neighbor SoSp-Routers only if required, then the listened neighbor SoSp-Routers react and periodically broadcast its beaconing with its ID. Within a limited time, each mobile device can accumulate the beaconing information from several SoSp-Routers and then decides the nearest SoSp-Router (by comparing the quality of beaconing (e.g., RSSI)) as its location. Additionally, the mobile device compares the new ID with old one and if changed, then the mobile sends the notify action for reporting new location to current SoSp-Router.

The above methods are used by the types of wireless communication protocols of the mobile devices in the heterogeneous environment consisting of various mobile and resource devices. These flexible localization algorithms provide significant scalability and availability in dynamic indoor circumstances.

## 3. Related Works

An indoor LBS system needs to increase the accuracy and precise localization of users or mobile devices as well as determine an efficient infrastructure topology in an intelligent building [12]. This key technology can be used to discover resources and to track target objects in a dynamic environment. In order to efficiently provide a dynamic indoor MAM system, a well-designed infrastructure architecture platform is needed.

An indoor LBS system, in conjunction with the Cisco Mobility Services Engine (MSE) [9] is a well-known commercial location system. The MSE estimates the number of visitors, the amount of time they spend, and the frequency of their visits within the site. Additionally, it provides knowledge of movement patterns by these visitors while they are in the building. However, in the MSE system based on a centralized architecture, a location server provides general location services for a network, and is primarily responsible for running the algorithms that predict client location. Therefore, the location server is also a single point of failure in the MSE system.

On the other hand, an approach towards a distributed architecture has also been suggested in related research. MobiEyes [13] presents a distributed architecture and a suite of optimization techniques for scalable processing of continuously moving location queries. The main idea behind the MobiEyes' distributed architecture is to promote a careful partition of a real-time location monitoring task into optimal coordination of server and client-side processing. In the MobiEyes system, the geographic area of interest is covered by several base stations, which are connected to the service provider's server. Then all location service requests are served through a three-tier architecture that consists of mobile objects, base stations, and the server. However, the MobiEyes system is not a fully distributed architecture, and even if the base stations act the part of multi-agents, a method of providing services based on a server system has a traffic congestion problem and a single point of failure in a dynamic environment with numerous mobile objects.

Real-world applications for an indoor LBS aim to detect the location of targets in various service domains, such as medical personnel or equipment in a hospital [14–17], museum guide [18], stored inventories in a warehouse [19], or tagged products in a supply chain [20]. In particular, many manufacturers like AeroScout [2], Ekahau [3], or Honeywell [21] support various commercial products for mobile asset management in a real-time location system (RTLS). Therefore, indoor MAM systems require technologies for location detection and real-time tracking of numerous mobile devices that have frequent mobility in the narrow space of a building.

In order to detect position, the various indoor MAM systems use a localization method based on WiFi wireless communication, as in several of the above works; however, many real-world RTLSs provide asset management solutions with a variety of signal types, such as a wireless local area network (WLAN), radio frequency identification (RFID), ultra-wide band (UWB), ZigBee, and so on. Various RTLS solutions have used these wireless techniques for management in a hospital environment with dynamic and frequent mobility and many assets.

Active RFID [13] containing a battery generally has a longer range than passive RFID, which has an extremely limited computational ability to assist applications and a very short read range. The simplest approach to localization using the RFID technique is to use proximity with readers. Asset applications for a hospital using RFID are well-used due to this relative simplicity.

However, these RTLSs with RFID have not been implemented on a large scale, and RTLS systems are more complex than RFID. Hence, a lot of research proposes RTLS systems combined with other wireless technologies, such as WLAN and WiFi [16]. These offer a number of different potential systems to exploit the benefits of RTLS for asset management in a hospital environment.

In these RTLS systems, tracking service requests for multiple mobile targets and from multiple monitoring devices can frequently occur. Therefore, an efficient distributed architecture for a dynamic environment is needed. We propose a platform based on self-organizing approach for tracking multiple mobile assets in an indoor MAM system. In addition, we also suggest a lookup algorithm and a multiple asset tracking method based on the proposed platform.

## 4. Proposed Architecture: SoSp Overview

### 4.1. Software Architecture of the SoSp-Router

**Figure 4.**
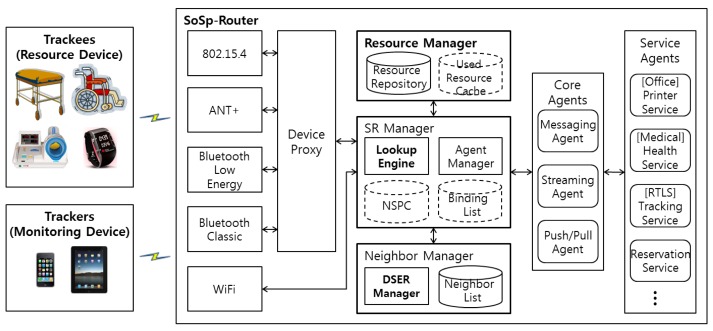
SoSp composition and SoSp-Router software architecture.

The proposed SoSp network architecture comprises SoSp-Routers that represent the unit space, resource devices used to provide physical services, and monitoring devices with which users request necessary services. Figure 4 shows the proposed software architecture of the SoSp-Router and the composition of the SoSp.

Resource devices such as health equipment have the capability of providing an LBS through wired/wireless communications with their SoSp-Routers, which are installed with communication modules in the unit space. A user can easily request any indoor MAM service from the physical resources (such as wheelchairs, people with a watch) with a monitoring device (such as a smartphone, or smart pad) using its embedded wireless communications function (e.g., WiFi, BLE, *etc.*). The monitoring devices and resource devices always communicate with the SoSp-Router in the unit space using LIDx & AMD [10] protocols, which provide real-time localization and the ability to transfer asynchronous messages amongst numerous mobile devices.

The internal software architecture of the SoSp-Router, which is the service agent and router for the distributed environment, comprises four components: the Device Proxy, the Resource Manager, the SoSp-Router (SR) Manager, and the Service Agents. The Device Proxy analyzes the communication data through the wireless communication module, after which it forwards the analyzed data to the requested Service Agent. This Device Proxy offers multifarious wireless communication protocols, including 802.15.4, BLE, and ANT+, but not WiFi protocol. Mobile devices using WiFi have the capability to communicate directly with the SR Manager; however, other mobile devices are capable of communicating through the Device Proxy.

The Resource Manager manages and stores the services and resources of the Service Agent running in the SoSp-Router in a Resource Repository. In addition, resources used more than once are stored in a Used Resource Cache (URC). This Resource Manager also manages the default configuration and the core resources for the SoSp-Router. The SR Manager enables inquiries of the requested service or resource with the help of the Resource Manager.

The SR Manager, working as the Agent Manager, controls the lifecycle of all Service Agents capable of running in the SoSp-Router. The mobile devices or resource devices with WiFi protocol directly interact with this SR Manager using the Ice [22] interface for a distributed platform. In addition, the SR Manager stores the list of neighboring SoSp-Routers and provides lookup results based on the proposed Lookup Engine, the details for which are discussed in the following section.

The Neighbor List consists of the physical neighbors connected through a geographical path, such as a door or a corridor, or logical neighbors grouped by a user's living spaces, such as a house or office. The distance values between the physical neighbors are calculated and determined by the mobile device traveling between the SoSp-Routers through the geographic path. The Neighbor Manager connects neighboring SoSp-Routers in the above manner and adds information about neighboring SoSp-Routers to the Neighbor List. In addition, the Dynamic Space Expanding and Reducing (DSER) Manager periodically sends a heartbeat message to all SoSp-Routers in the Neighbor List to detect a removed neighboring SoSp-Router from the current infrastructure, and provisionally has its neighbors' neighbor lists for responding.

Finally, the Service Agents, which are related to the service resources and service applications for the SoSp-Router, manage the requested service data and communicate with resource devices through the wireless communication module in the unit space. There are core Service Agents managed by the Resource Manager, and core services can always be provided in the current SoSp-Router. These core services have particular characteristics depending on each individual SoSp-Router installed in various locations. In order to provide various services in the Service Agents, the SoSp-Router needs the core functions to support the requested services. For example, in a medical service system, patients or staff members can request health data anywhere and anytime. In order to send the requested health data to the user, a messaging function is always required in the SoSp-Router middleware, or a streaming function is required for continuously receiving health data from wearable medical equipment. And, in order to send and receive the data anywhere and anytime, a push/pull function is needed. In addition, these core functions must be always supported.

The proposed SoSp consists of the SoSp-Router, resource devices, and mobile devices in an overlay network platform of a two-tier architecture (*i.e.*, stationary nodes and mobile nodes). This concept of the SoSp has been adapted for a self-organizing and fully distributed network infrastructure providing scalability and availability, unlike a centralized server-based architecture.

### 4.2. Operation Principles of the Lookup Engine

Each SoSp-Router has a unique identifier, an IP address, as well as a proxy for Ice objects besides group and place, and this information regarding neighboring SoSp-Routers is stored with distance values in the Neighbor List. The information on neighboring SoSp-Routers includes the distance values from the current SoSp-Router calculated by a mobile device according to the above method to create an overlay network map. The Lookup Engine in the SoSp-Router discovers requested resources based on the neighbor lists of connected SoSp-Routers.

**Figure 5.**
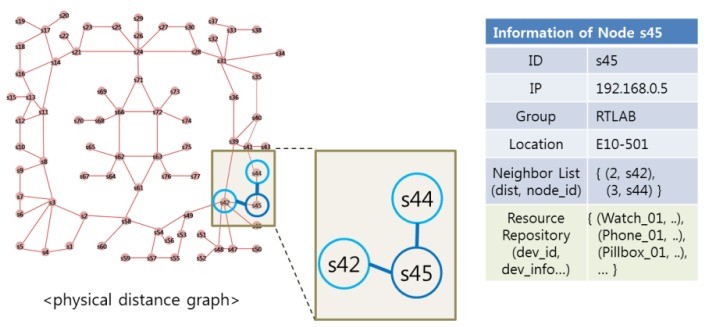
SoSp-Router with the information about neighboring SoSp-Routers.

As shown in Figure 5, each SoSp-Router only knows the distance and information of physical neighboring SoSp-Routers. This information is used to create the neighbor shortest path cache (NSPC) in each SoSp-Router. In addition, the SoSp-Router manages mobile or stationary resource devices such as watches, phones, or pill-boxs within range of the unit space. The proposed NSPC is created and extended using the neighbor lists of each SoSp-Router. Initially, the Lookup Engine creates an NSPC, which has as local information distance values of 0. Next, this NSPC is extended with every lookup attempt.

Indoor MAM via mobile devices has to be provided within the service available range, which is mostly close to the mobile device user's location. The service available range depends on the service characteristics and is set to constraint conditions to provide the indoor MAM. For example, service for a physical resource, such as a wheelchair or an infusion pump, needs a relatively small area. However, if a mobile phone user in an office requires medical data at a house or hospital, constraint conditions for authorization (such as available group, user, and authenticated device) are highly significant, compared to the service range. Another constraint is data about a logical group, which are defined by the location installed. This logical group constraint determines whether a mobile user can or cannot use the resource service within range of the SoSp-Router. In addition, information about the resource service contains the list of enabled mobile devices. In other words, each mobile user is authenticated by this constraint. The service and lookup information must include these constraint conditions and data. This information is used to discover and manage the various service resources by the Lookup Engine. The lookup information also includes two constraints of the above conditions for the logical group or max range.

**Figure 6.**
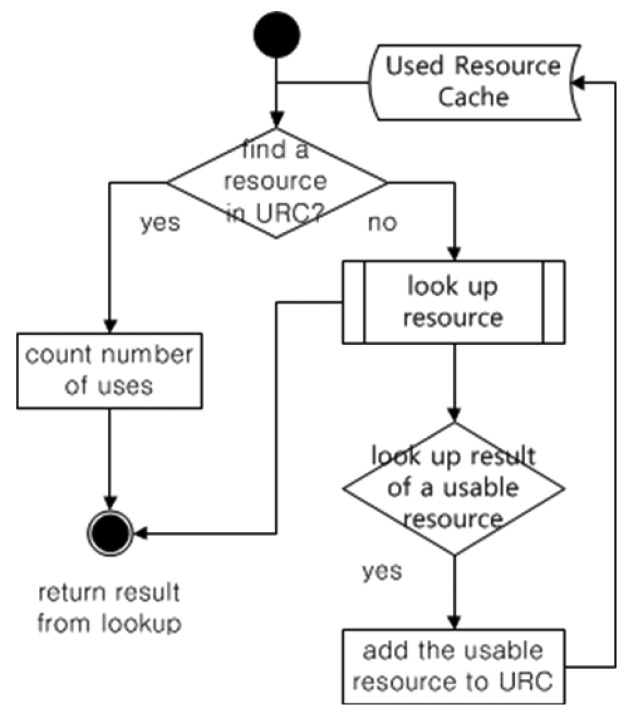
The flow diagram of the URC management.

In addition, the proposed lookup procedure includes a cached lookup history, which is the Used Resource Cache (URC) managed by the Resource Manager. The service resources searched and used in the above lookup manner are stored in the URC as shown in Figure 6. The used resources are stored and counted when these are reused in the URC. If the stored resource in the URC is not used anymore, the resource is no longer managed. Hence, the proposed architecture is highly efficient as time passes due to the fact that its lookup history is cached.

## 5. Tracking Multiple Mobiles Based on SoSp

### 5.1. Lookup Mobile Devices for Tracking Service

Initially, the SN Manager configures and initializes the default information of a SoSp-Router for connecting with neighboring SoSp-Routers, after which it commences the core services, which are registered in the Resource Repository of the Resource Manager, and which include the Service Agents for the service event opportunities occurring for any particular person or device. The resource devices, which enable bidirectional communication with the Device Proxy in each SoSp-Router through WiFi or different RF protocols such as 802.15.4, ANT+, or BLE, are capability of transferring and receiving data as well as requesting services. The SN Manager searches for an appropriate Service Agent and forwards a connectable Service Proxy to the resource device or Device Proxy, making the requested services practicable. If the requested practicable Service Agent is not running, the SN Manager launches this service and forwards the proxy object of this Service Agent to the mobile device. After the mobile device acquires the proxy object on demand, it is directly connected with the relevant Service Ageant.

**Figure 7.**
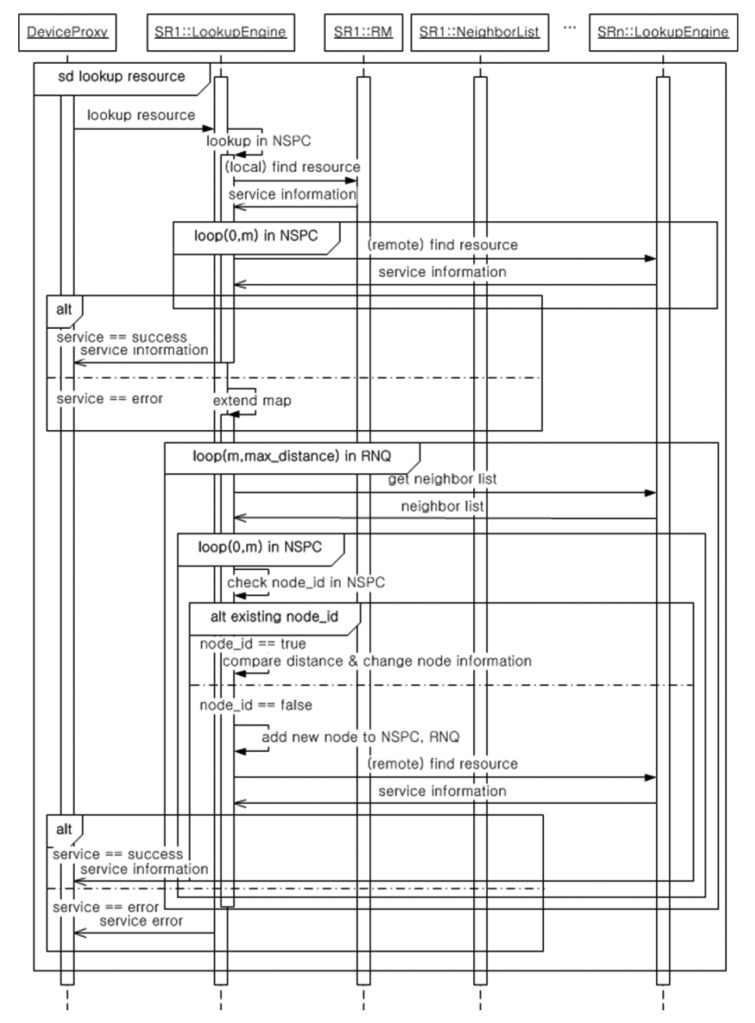
The sequence of indoor LBS lookup.

Figure 7 shows the detailed procedure of the Lookup Engine in the SR Manager used to discover the available resources of the nearest physical neighbor to the current position of the mobile device. The lookup sequence consists of two parts. First, the Lookup Engine with an NSPQ discovers the requested resource from the Device Proxy in the neighboring SoSp-Router based on the current NSPQ. If there are no requested resources in the Neighbor List based on the current NSPQ, the NSPQ is extended to search for resources in the next neighbor SoSp-Router. This process repeats until the total distance to the neighboring SoSp-Router meets the conditions of maximum constraint.

The proposed lookup manner using the search history cache quickly obtains lookup results with the exception of the initial lookup process, which is mainly still mainly in the extend lookup phase. In particular, the proposed fully distributed architecture is relatively stable compared to a centralized manner whenever numerous mobile devices concurrently request service lookups from several locations.

### 5.2. Tracking Procedure in the SoSp

In an RTLS system such as a complex and dynamic hospital environment, tracking service requests by multiple mobile trackees and trackers can frequently occur. For instance, a doctor requires looking up his or her patients, and other staff in the hospital also desires to monitor them at the same time. Therefore, the RTLS system for multiple mobile tracking in real time necessitates the specific architecture and method.

**Figure 8.**
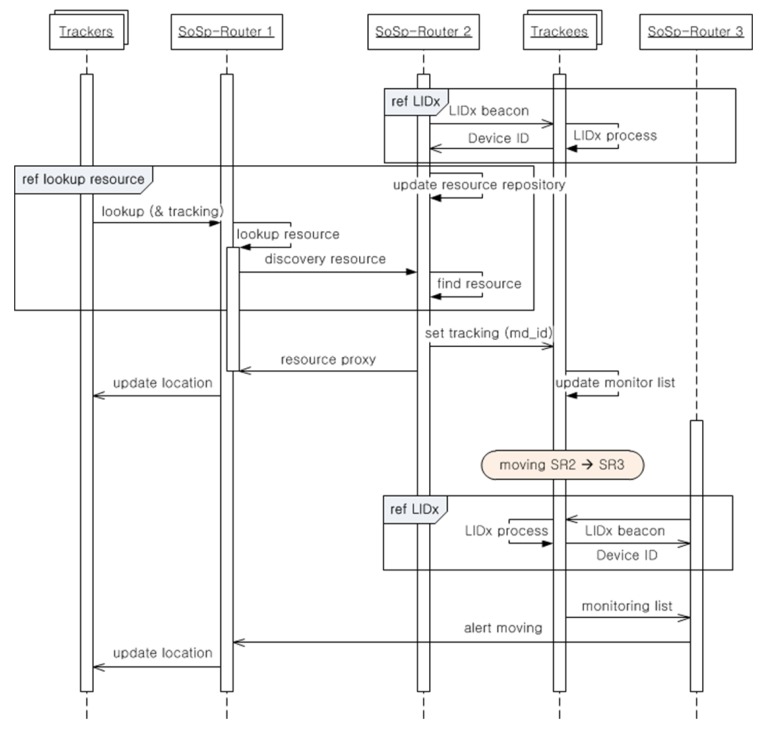
The tracking service process.

Figure 8 describes the process whereby multiple trackers look up and check the location of multiple moving trackees attached to the equipment or possessed by patients in the proposed infrastructure and confined to the SoSp-Router built onto the ceiling in each room or corridor. In the shown sequence, trackers are the mobile devices such as smart phones or smart pads with WiFi wireless communication functions, and resource devices include people with a watch or tag including the RF wireless communication module, or medical equipment, such as beds, wheelchairs, or infusion pumps, with continual or intermittent mobility. Of course, the RF wireless module is attached to the medical equipment for real-time localization and communication with the SoSp-Router or other devices.

All of the resource devices in the range of a SoSp-Router are always localized using the LIDx [10] protocol to exchange IDs between both SoSp-Router and resource device. In order to start a tracking service, the monitoring device requests the lookup resource device from the connected SoSp-Router. The SoSp-Router finds the resource device using the lookup algorithm based on the Lookup Engine and processes the request depending on the type of resource. And then, the SoSp-Router representing the position of the needed resource device sends to the target resource device a message setting the tracking service with the ID of the monitoring device. The resource device that receives the tracking message creates a new monitoring list or updates the current monitoring list. The monitoring device that receives the position of the target resource device from the SoSp-Router refreshes the current monitoring display. If the target resource device is moved from, for example, SoSp-Router 2 to SoSp-Router 3, SoSp-Router 3 also runs the LIDx process for localization. The resource devices send the current monitoring list with the ID of the monitoring device to SoSp-Router 3. SoSp-Router 3 receives the list of monitoring devices and alerts the movement of the resource device to within its range.

**Figure 9.**
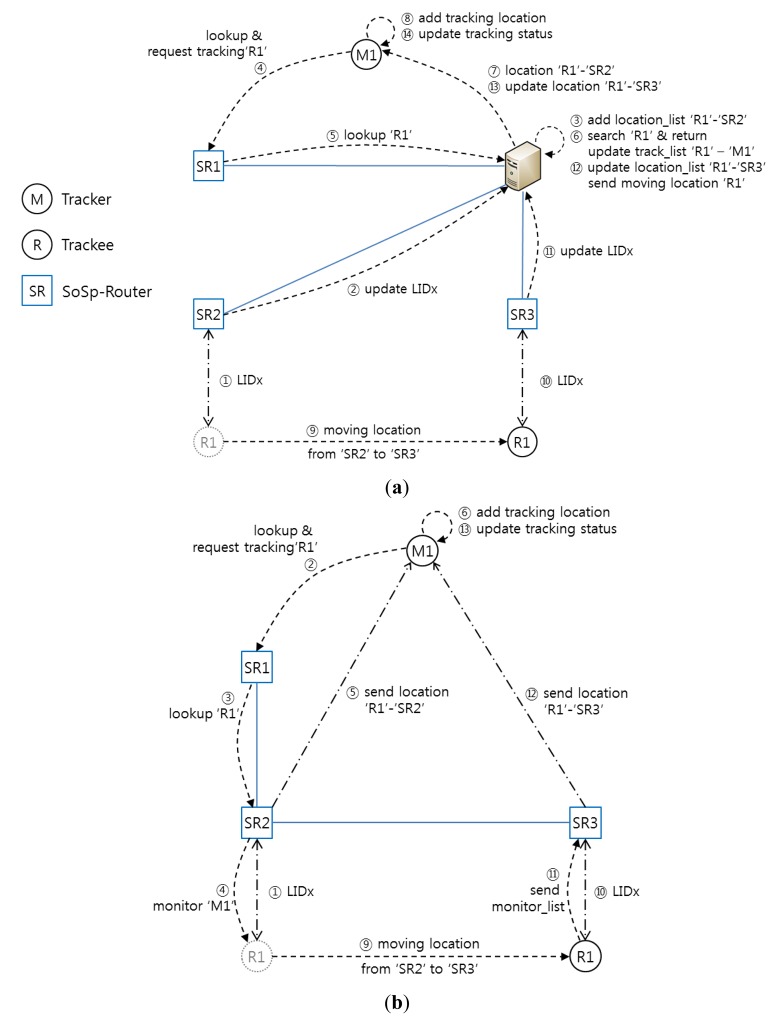
The tracking service process in a centralized platform and the proposed self-organizing distributed platform. (**a**) Tracking state diagram in a centralized platform; (**b**) Tracking state diagram in the proposed SoSp.

Figure 9 shows a detailed comparison of the proposed self-organizing distributed platform and a centralized platform. First, Figure 9a illustrates the tracking state diagram in a centralized platform. (1) SoSp-Router ‘SR2’ recognizes that resource device ‘R1’ is within its range via the LIDx protocol. (2) The centralized service-based platform needs a report about the position of each resource device. (3) The location server system adds or updates the current position of resource device ‘R1’ in the location database. (4) If monitoring device ‘M1’ needs the tracking service for resource device ‘R1’, it requests the lookup and tracking service for the target resource connected to SoSp-Router ‘SR1’. (5) SoSp-Router ‘SR1’ sends the lookup resource message regarding ‘R1’ to the location server system. (6) And then, the server searches for resource ‘R1’ in the location database and updates the current tracking list. (7) The server sends the current position of resource ‘R1’ to the monitoring device. (8) Monitoring device ‘M1’ then receives the location message for the target resource device and draws the position on its current monitoring display and updates the status of ‘R1’. (9) If, while providing the tracking service, resource device ‘R1’ moves into range of SoSp-Router ‘SR3’, (10) the SoSp-Router and resource device perform the location positioning process using LIDx protocol. (11) SoSp-Router ‘SR3’ reports the new position of resource ‘R1’ to the location server. (12) The location server updates the database, and checks the tracking list. (13) If ‘R1’ is included in the tracking list, the server alerts the movement of resource ‘R1’. (14) And then, monitoring device ‘M1’ refreshes the position of resource ‘R1’. If there are multiple trackers and trackees, the same sequence applies equally. This process in the centralized platform has a single point of failure as shown in Figure 9a. In addition, because the central server has the centralized location database and tracking list, traffic congestion can occur when movement of numerous resource devices occurs concurrently at several different points.

On the other hand, in the proposed self-organizing distributed platform, data transmission only occurs between the monitoring devices and the currently positioned SoSp-Router of the target resource device. Comparing the Figure 9), it appears the proposed self-organizing distributed platform has an uncomplicated and uninvolved process compared to the centralized platform. This feature shows the proposed platform is highly efficient when multitudinous tracking services are concurrently present and when numerous resource devices have frequent mobility at several locations.

## 6. Implementation and Evaluation

### 6.1. The SoSp-Router and Mobile Device Hardware/Software Implementation

SoSp-Routers are installed in every unit space of a building, such as a room or a corridor with a wired communication protocol such as Ethernet and various wireless communication protocols such as WiFi, 802.15.4, BLE, or ANT+.

**Figure 10.**
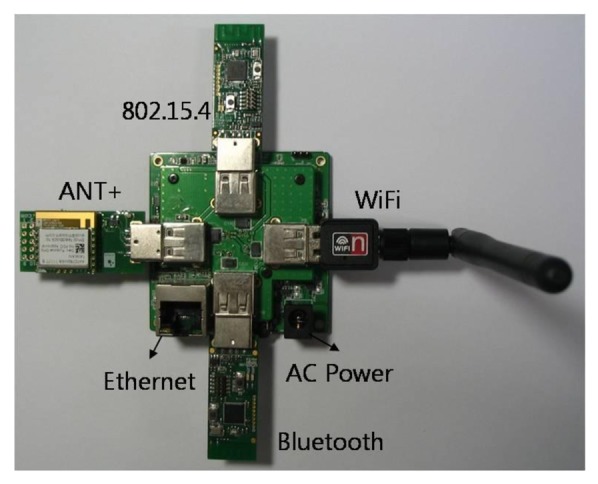
The SoSp-Router hardware with its various wireless communication modules.

The SoSp-Router hardware, as shown in Figure 10, is designed to accept various wireless communication modules using USB ports. The SoSp-Router utilizes a wired 10/100 Ethernet communication port for the constituent infrastructure of the indoor MAM system as well as four USB 2.0 ports for wireless communication modules.

In order to attain the cooperation needed between the neighboring SoSp-Routers and mobile devices using the wired/wireless networks in a self-organizing and fully distributed environment, the internal software and smartphone application components are implemented using Ice [22], a distributed platform middleware. Ice facilitates the development of heterogeneous distributed applications by supporting various languages, such as C++, Java, C#, Python, Object-C, Ruby, and PHP over Windows, Linux, iOS, and Android platforms.

**Figure 11.**
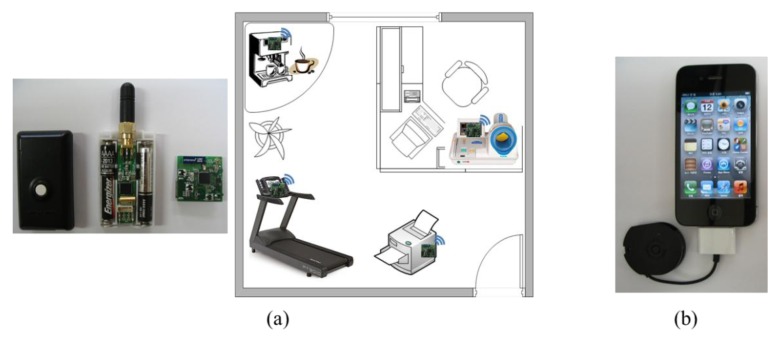
Service resource devices and mobile devices owned by users. (**a**) Trackees (service resource devices) with communication module; (**b**) Tracker (mobile device).

There are various types of cell-based SoSp-Routers and mobile devices using wireless communications in the infrastructure. Resource devices, such as office equipment, home appliances, and health equipment in the unit space, can provide real-time localization using LIDx & AMD [10,11] wireless communication protocol developed by our research team. Mobile devices use diverse types of developed dongles for smartphones besides watches, smartphones, or smart pads. Mobile devices provide an adaptive service using an appropriate profile for attributes of the devices through the connection with the resource devices. In particular, ID matching with a mobile device owned by a user is important for resources requiring privacy, such as medical and health equipment; users launch a request to a service using their individual mobile devices. Figure 11 shows the communication modules built into the resource devices and various types of mobile devices owned by users.

### 6.2. SoSp-Router Tests and Evaluation in Real Test Bed

**Figure 12.**
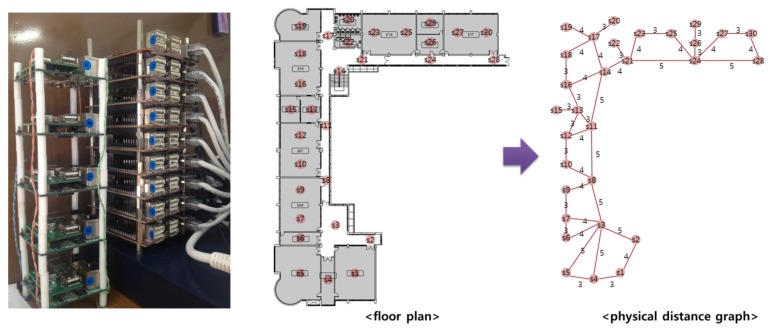
Real test-bed with SoSp-Router boards, and the floor plan of the test bed layout.

Each SoSp-Router is attached to the ceiling in a unit space, such as a room or a corridor, and represents the position located within its range. The SoSp-Router board has an ARM Cortex A8 MCU with 512MB SDRAM, an Ethernet port for connection with neighboring SoSp-Routers, and an IEEE 802.15.4 transceiver for communication with mobile nodes. In the real test bed evaluation, we set up a test bed with 15 SoSp-Router boards, which had two lookup processes each, and composed a physical distance graph comprising 30 nodes from the floor plan, as shown in Figure 12.

Each SoSp-Router has more than one process, including a lookup process, in practice. In order to evaluate a large number of lookups from numerous mobile devices, the simulation program sent numerous requested service packets to each lookup process in the SoSp-Router board, similar to the preceding simulation test.

**Figure 13.**
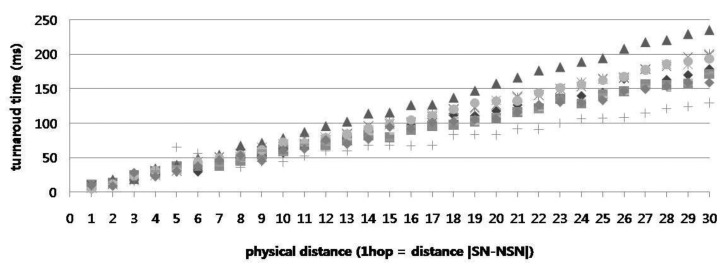
Turnaround times by distance for one SoSp-Router.

In the real test bed, the services or resources requested by the user are generally located in close proximity to the user's current position. Therefore, the proposed Lookup Engine (based on the geographically shortest path, not a path by network hop count) is highly efficient in reality. Figure 13 shows the turnaround times by distance value for one SoSp-Router; the response time in the SoSp-Router is directly proportional to the distance to the neighbor SoSp-Router, similar to the preceding simulation results. In the real test bed, this result indicates that the proposed Lookup Engine with the NSPC is adequate for providing an indoor MAM.

**Figure 14.**
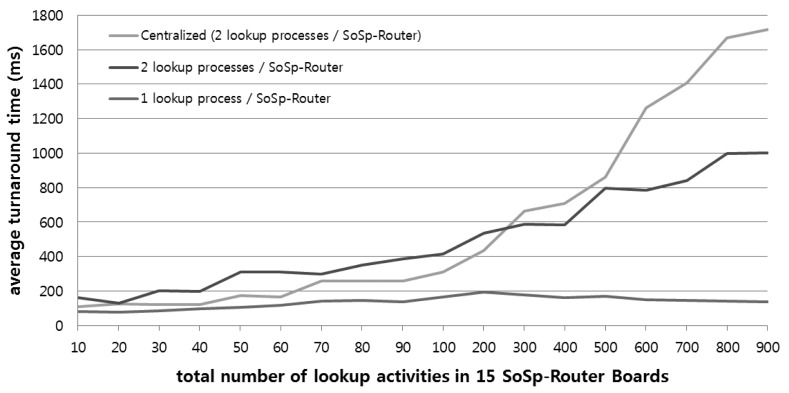
Comparison of average turnaround times for concurrent searches among all 30 lookup processes in the real test bed environment.

Figure 14 shows a graph that compares average turnaround times for the lookup response between the proposed architecture and a traditional centralized architecture when numerous mobile devices concurrently request service lookup from several locations. We randomly created the service lookups activities from 10 to 900 in all of the lookup processes in 15 SoSp-Router boards. Since one SoSp-Router mostly has more than one process, including the lookup process, in practice, we set two test conditions: one lookup process or two lookup processes in one SoSp-Router board. As shown in Figure 14, the test results using two lookup processes had network and processing overhead, compared with conditions with one lookup process. However, in cases where the total lookup number is under 300, the centralized architecture is faster than the proposed architecture. The real test bed performance of the proposed architecture grows steadily upon increasing the lookup service requests. However, the centralized architecture result rises dramatically under the same circumstances.

### 6.3. Multiple Devices Tracking Test and Evaluation

**Figure 15.**
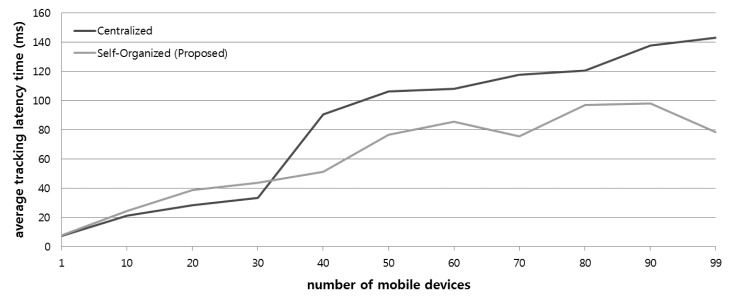
Comparison of average tracking latency times from movement based on the number of mobile resource devices.

In the tracking service system, the latency time when updating location positions of target mobile devices is an important factor. In particular, latency increases with frequent and dynamic mobility of numerous mobile devices. In order to evaluate the performance of this factor in the tracking architecture based on the proposed platform, Figure 15 shows a graph that compares the proposed architecture to the traditional centralized architecture for average latency from updating the movement of target devices when numerous mobile devices concurrently move from several locations.

We created 1 to 100 resource devices in each SoSp-Router, and translocated the position of each mobile device to a random SoSp-Router. And then, 10 monitoring devices requested the start of the tracking service for all mobile devices, and received the movement messages of the mobile devices to update their location status. We measured the tracking latency time between moving mobile devices and updating monitoring devices in one system that can simulate the behavior of monitoring and moving via the LIDx protocol within a period of 1sec.

As shown in Figure 15, when the total mobile device number is under 30, the centralized architecture has lower latency than the proposed architecture. However, the performance of the proposed architecture shows comparatively lower results with an increasing number of mobile devices. This evaluation result shows that the centralized architecture is the single point for concentration of traffic from numerous mobile devices.

In order to evaluate the traffic concentration, we measured the sending and receiving of data traffic in each SoSp-Router. We created 10 monitoring devices and 100 mobile resource devices, and each monitoring device requested the tracking service for all mobile resource devices. All mobile resource devices have movement of 1 s intervals during 5 min among a total of 9 SoSp-Routers. In the centralized platform, the location and tracking management server process is only added under the same circumstances of the proposed platform, and each SoSp-Router only forwards packet data as shown in Figure 9.

**Figure 16.**
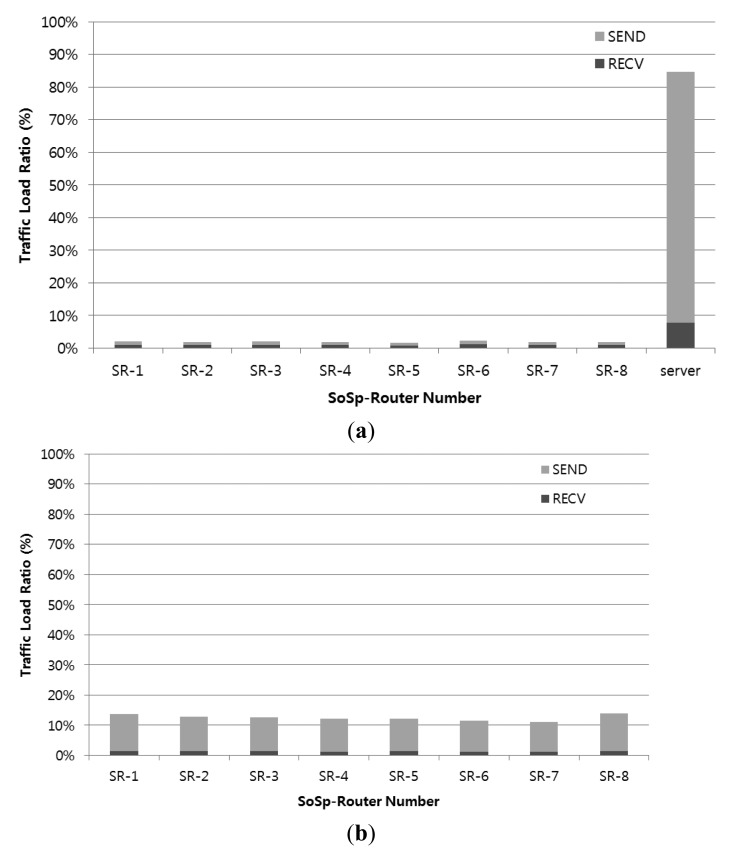
Traffic load ratio in each SoSp-Router and server. (**a**) Traffic load ratio in the centralized architecture; (**b**) Traffic load ratio in the proposed self-organizing architecture.

Figure 16 shows two graphs comparing the traditional centralized architecture and the proposed self-organizing architecture. In the proposed platform, the traffic load ratio was evenly distributed among all SoSp-Routers, whereas the data flow converged towards the server in the centralized platform. This result shows that the centralized architecture has a single point of failure, and traffic congestion can occur when numerous mobile devices concurrently have frequent and dynamic mobility.

## 7. Conclusions

This paper proposes a self-organizing and fully distributed indoor MAM platform and architecture for tracking multiple mobiles and monitoring devices based on the proposed platform. The proposed self-organizing overlay network is built on top of the legacy network within a geographic distance, and the SoSp-Router represents a unit space built to compose the infrastructure of the overlay network. A self-organizing approach from nature is applied to the proposed platform, which has a simple and regionalized infrastructure. Each SoSp-Router only knows information about direct neighboring SoSp-Routers connected through a physical path such as a door or corridor without a centralized server system. This cell-based SoSp-Router determines the necessary services and resources using only information regarding its neighbors through cooperation amongst neighboring SoSp-Routers.

In particular, a tracking service for multiple trackers and trackees in a dynamic hospital environment is necessary for an efficient architecture that reflects a different approach to the traditional centralized platform. In a dynamic hospital environment, numerous mobile assets such as wheelchairs and infusion pumps are continuously relocated throughout the hospital, and many patients and staff also frequently move throughout the hospital. This paper suggests a tracking service architecture based on the proposed self-organizing and fully distributed platform.

We also determined that the proposed Lookup Engine with NSPQ is highly efficient over time or as the number of lookups increases through performance evaluation in a real test bed configured with real SoSp-Router boards. In addition, we evaluated tracking latency and the traffic load ratio in the proposed tracking architecture, and showed that the proposed architecture is more efficient than the traditional centralized platform when providing a tracking service for numerous mobile devices. The proposed SoSp platform with SoSp-Routers enhances scalability, decentralization, fairness, and robustness whenever numerous mobile devices concurrently request resource lookups from several locations. In addition, there is no need to maintain a map of the entire indoor location, unlike traditional centralized server methods.

Future research will focus on extending the advanced indoor MAM applications. For example, a medical streaming system can consistently send ECG waveform data despite patients' frequent mobility. Additionally, we are studying a dynamic space that expands and contracts with a self-organizing approach in a dynamic indoor environment. If some SoSp-Routers are temporarily deleted or reactivated, a service is not supported in a distributed architecture system. The dynamic service space will provide the reliable service using a redirected path. Therefore, the additional study will make the proposed system is more robust.

## References

[1] Bellavista P., Kupper A., Helal S. (2008). Location-based services: Back to the future. Pervasive Comput..

[2] WiFi Based RTLS Solutions & Wireless Sensor Technologies by Aeroscout. http://www.aeroscout.com/.

[3] Kuroda T., Sasaki H., Suenaga T., Masuda Y., Yasumuro Y., Hori K., Ohboshi N., Takemura T., Chihara K., Yoshihara H. (2012). Embedded ubiquitous services on hospital information systems. IEEE Trans. Inf. Technol. Biomed..

[4] Cupper A., Treu G., Linnhoff-Popien C. (2006). TraX: A device-centric middleware framework for location-based services. IEEE Commun. Mag..

[5] Ruppel P., Klein C., Linnhoff-Popien C. Indooria—A Platform for Proactive Indoor Location-Based Services.

[6] Lua E.K., Crowcroft J., Pias M., Sharma R., Lim S. (2004). A survey and comparison of peer-to-peer overlay network schemes. IEEE Commun. Surv. Tutor..

[7] Conti M., Kumar M. (2010). Opportunities in opportunistic computing. Computer.

[8] Zhu F., Mutka M.W., Ni L.M. (2005). Service discovery in pervasive computing environments. IEEE Pervasive Comput..

[9] Wi-Fi Location-Based Services 4.1 Design Guide—Cisco Systems. http://www.cisco.com/en/US/docs/solutions/Enterprise/Mobility/WiFiLBS-DG.html.

[10] Kim T.H., Jo H.G., Lee J.S., Kang S.J. (2012). A Mobile Asset Tracking System Architecture under Mobile-Stationary Co-Existing WSNs. Sensors.

[11] Lee D.K., Kim T.H., Jeong S.Y., Kang S.J. (2011). A three-tier middleware architecture supporting bidirectional location tracking of numerous mobile nodes under legacy WSN environment. J. Syst. Archit..

[12] Gressmann B., Klimek H., Turau V. Towards Ubiquitous Indoor Location Based Services and Indoor Navigation.

[13] Gedik B., Liu L. (2006). MobiEyes: A Distributed Location Monitoring Service Using Moving Location Queries. IEEE Trans. Mobile Comput..

[14] Boulos M.N.K., Berry G. (2012). Real-time locating systems (RTLS) in healthcare: A condensed primer. Int. J. Health Geogr..

[15] Mun I.K., Kantrowitz A.B., Carmel P.W., Mason K.P., Engels D.W. Active RFID System Augmented With 2D Barcode for Asset Management in a Hospital Setting.

[16] Schrooyen F., Baert I., Truijen S., Pieters L., Denis T., Williame K., Weyn M. (2006). Real Time Location System over WiFi in a Healthcare Environment. J. Inf. Technol. Healthc..

[17] Youna J.H., Alia H., Sharif H., Deogun J., Uher J., Hinrichs S.H. WLAN-Based Real-Time Asset Tracking System in Healthcare Environments.

[18] García O., Alonso R.S., Guevara F., Sancho D., Sánchez M., Bajo J. (2011). ARTIZT: Applying Ambient Intelligence to a Museum Guide Scenario. Ambient Intelligence—Software and Applications.

[19] Ding B., Chen L., Chen D., Yuan H. Application of RTLS in Warehouse Management based on RFID and Wi-Fi.

[20] Martinez-Sala A.S., Egea-Lopez E., Garcia-Sanchez F., Garcia-Haro J. (2009). Tracking of Returnable Packaging and Transport Units with active RFID in the grocery supply chain. Comput. Ind..

[21] Honeywell Building Solutions—NASIA. https://buildingsolutions.honeywell.com/.

[22] Henning M., Spruiell M. Distributed Programming with Ice, ZeroC. http://www.zeroc.com/Ice-Manual.pdf.

